# Reduced expression of human metapneumovirus matrix protein impacts virus assembly and ribonucleoprotein localization

**DOI:** 10.1128/jvi.01066-26

**Published:** 2026-08-31

**Authors:** Chase Jordan Heim, David Adam Stein, Hong M. Moulton, Emilia Galperin, Rebecca Ellis Dutch

**Affiliations:** 1 Department of Molecular and Cellular Biochemistry, College of Medicine, University of Kentucky 167091, Lexington, Kentucky, USA; 2 Department of Biomedical Sciences, Gary R. Carlson, MD, College of Veterinary Medicine, Oregon State University 574476, Corvallis, Oregon, USA; St Jude Children's Research Hospital, Memphis, Tennessee, USA

**Keywords:** human metapneumovirus, matrix proteins, virus assembly, NNSVs

## Abstract

Human metapneumovirus (HMPV) causes severe respiratory tract infections in all cohorts, particularly in vulnerable groups such as children, older adults, and the immunocompromised. The matrix (M) protein of HMPV, like the M proteins of other members of the order *Mononegavirales*, is involved in virus assembly and budding. However, other functions of the HMPV M protein have yet to be elucidated. To investigate the various functions of the M protein, we used a peptide-conjugated phosphorodiamidate morpholino oligomer (PPMO) antisense agent designed to block translation of the M gene during infection. Treatment with the PPMO targeted against M mRNA (PPMO_M_) led to dose-dependent decreases in M expression, and a 10 µM PPMO_M_ treatment applied at 1 hour post-infection (hpi) led to a >95% reduction in M protein at 24 hpi. Reductions in M expression had a significant impact on virus production and viral titers. Fluorescence *in situ* hybridization (FISH) probes used to detect vRNA showed that a reduction in M led to an increase in inclusion body (IB) count per cell, but a decrease in the average IB volume per cell at 12 and 24 hpi. HMPV-infected cells treated with PPMO_M_ exhibited lower levels of phosphoprotein (P) at the plasma membrane. Importantly, no change in viral ribonucleoprotein movement (vRNP) was observed when the M protein levels were reduced. These studies support a model where the M protein does not facilitate movement, but instead is critical for the transfer to and stable association of vRNPs with the assembly sites at the membrane.

**IMPORTANCE:**

Human metapneumovirus (HMPV) is a respiratory pathogen that can cause severe infections in humans, but there are no targeted treatment options for HMPV. To better characterize HMPV, a matrix (M) protein-targeted peptide-conjugated phosphorodiamidate morpholino oligomer (PPMO_M_) was designed to control M expression during infection. Inhibition of M expression resulted in infections with reduced viral filaments and less viral RNA at the cell periphery. A reduction in M protein expression did not disrupt the active transport of viral ribonucleoproteins (vRNPs) but did significantly decrease vRNP localization at membranes and increase the number of inclusion bodies within infected cells. Viral titers were significantly reduced when M protein expression was inhibited, and dose-dependent reductions in M protein directly correlated with viral particle production. This study uses a unique tool to characterize the HMPV M protein during infection.

## INTRODUCTION

Human metapneumovirus (HMPV) is a common respiratory virus in the *Pneumoviridae* family that can cause severe infections in all individuals, particularly in vulnerable groups such as children, older adults, and the immunocompromised (1–3). A meta-analysis showed that the global prevalence of HMPV among patients with respiratory tract infections was 5.3% (4). Another study estimated that there were 14.2 million global HMPV-associated respiratory tract infections in children younger than 5 years old in 2018 (5), while the respiratory syncytial virus (RSV) had an estimated 33 million cases in 2019 (6).

HMPV is a non-segmented negative-sense RNA virus (NNSV) with a ~13.3 kb genome that contains eight genes coding for nine proteins (7–10). The fusion (F), attachment (G), and small hydrophobic (SH) glycoproteins reside in the lipid envelope of viral particles and have roles in virus entry and membrane permeability (11–14). The nucleoprotein (N), phosphoprotein (P), large polymerase (L), and M2 proteins are involved in viral replication and transcription (15–17), although M2-1 and M2-2 are dispensable for HMPV infection in cell culture (18–20). The matrix (M) proteins of viruses in the order *Mononegavirales* have been widely studied for their role in virus assembly and budding (21, 22). Briefly, virus assembly begins with the localization of M, P, and F at the plasma membrane of cells, which then drives budding in the form of viral filaments (23, 24). Other functions of the M protein for related viruses have been suggested, including nuclear localization (17, 25–32).

A critical role for the M protein in budding has been shown for many NNSVs, and it is clear that dimerization and lipid interactions are critical for this function (21, 33, 34). M proteins have also been shown to localize to sites of viral replication called inclusion bodies (IBs), potentially through interactions with other proteins, such as F, M2-1, and P (17, 22, 23, 35). An RSV F protein containing cytoplasmic-tail truncations resulted in the accumulation of the M and F proteins at IBs, suggesting that the F cytoplasmic tail recruits viral ribonucleoproteins (vRNPs), composed of N, viral RNA (vRNA), P, and L, at IBs and interacts with the M protein (22). The RSV M protein has also been shown to interact with three sites of the RSV P protein, which is likely important for the formation of viral filaments when M and P are combined with the F protein (23). Taken together, vRNP trafficking and budding may depend on M and its interactions with other viral proteins; however, some of the functional implications of these interactions are less clear, such as intracellular trafficking.

Several prior studies of *Mononegavirales* M proteins have utilized recombinant viruses containing a mutated or deleted M gene. Infection with M-null RSV abolished the formation of long viral filaments on the surface of infected cells; however, shorter filaments with the F and G proteins were still present (36). Another study that removed the M gene from the Nipah virus genome showed its important role in viral budding and particle stability (37). Completely knocking out the M gene does have experimental limitations, as some amount of M may need to be present for viral propagation. Additionally, current experimental systems provide no mechanism to control how much M protein is expressed at various times during an infection. Specific reductions in M protein expression that could be controlled, at least to some extent, and initiated at different stages of infection could provide novel insights. Therefore, we utilized an antisense peptide-conjugated phosphorodiamidate morpholino oligomer (PPMO) to knock down M expression at the mRNA translation stage. PPMOs have previously been used to successfully block the translation of specific mRNAs in various viruses (38–44); however, they have not been used to produce varying expression levels of specific proteins at different time points during an infection.

We show that a PPMO targeting HMPV M mRNA specifically reduces M expression during infection in a dose-dependent manner. We confirmed that the M protein is critical for producing authentic virus and that M expression levels directly correlate with viral particle production. Immunofluorescence staining in cells with reduced M expression showed a lack of mature viral filaments, indicating that the HMPV M protein is important for the maturation of assembly sites. We also show that the F protein had a diffuse staining pattern on membranes and that the N protein failed to localize to these areas when the M protein was reduced. We observed changes in vRNA localization using fluorescence *in situ* hybridization (FISH) when M was reduced, including decreased vRNA localization at the cell periphery. This suggests that the M protein is involved in vRNA transport to assembly sites, and experiments tracking mCherry-tagged P (mCherryP), a marker for vRNP complexes, showed a significant decrease in vRNP localization at the plasma membrane when M was reduced. Live-cell imaging showed that vRNPs move in the cell with M, but there was no change in the speed or number of vRNP movements when M was significantly reduced. These data further support that the M protein is involved in vRNP localization and that it facilitates the transfer of vRNPs from RNP-carrying endosomes to assembly sites.

## MATERIALS AND METHODS

### Cell lines, viruses, and antibodies

Lung carcinoma (A549) cells were obtained from ATCC (CCL-185) and grown in Ham’s F12, Kaighn’s Modification (Cytiva/HyClone) supplemented with 10% heat-inactivated fetal bovine serum (FBS; Sigma-Aldrich). African green monkey kidney (Vero) cells were obtained from ATCC (CCL-81) and cultured in DMEM (Cytiva/HyClone) supplemented with 10% heat-inactivated fetal bovine serum (FBS; Sigma-Aldrich). Wild-type (WT) HMPV CAN 97-83 (a gift provided by Guy Boivin, Université Laval, Canada) and recombinant, GFP-expressing HMPV (rgHMPV) CAN 97-83 (a gift provided by Peter Collins and Ursula Buchholz, NIH, USA) were propagated in Vero cells as previously described (45). Briefly, Vero cells were plated to be 70%–90% confluent and infected with HMPV at a multiplicity of infection (MOI) of 0.01. The virus propagation was supplemented with 200 µM L-glutamine and 0.3 µg/mL TPCK-Trypsin daily until severe pathology was observed. The cells were scraped and harvested with 10× sucrose phosphate glutamate buffer for storage at −80°C. The propagation was freeze-thaw-cycled a total of three times and clarified at 2,500 × *g* for 10 min at 4°C using a Thermo Fisher Scientific Sorvall ST 8R centrifuge. The supernatant was transferred onto a 20% sucrose in 1× TNE (150 mM NaCl, 50 mM Tris-HCl, 1 mM EDTA, pH 7.5) cushion and spun at 131,500 × *g* using a SW28 rotor on the Optima XPN-90 Ultracentrifuge for 2.5 h at 4°C and resuspended in OptiMEM. Titers were calculated by serially diluting virus media and infecting A549s, then staining for infected cells with a mouse anti-HMPV F antibody, and analyzed by flow cytometry or immunofluorescence. When purifying the virus for immunoblotting, the culture supernatant was collected and clarified at 2,500 × *g* for 10 min at room temperature. The clarified supernatant was then loaded onto a 20% sucrose in 1× TNE cushion and centrifuged at 128,928 × *g* using the Sorvall Discovery M120 ultracentrifuge for 1 h at 4°C. The purified virus was resuspended with 1× SDS denaturing loading buffer (20% glycerol vol/vol, 1.5% DTT wt/vol, 30 mM Tris-HCl pH 6.8, 20% SDS wt/vol). Antibodies used in this study are mouse anti-HMPV N (Bio-Rad), mouse anti-HMPV F (Abcam), mouse anti-HMPV M (Thermo Scientific), rabbit anti-M (kindly provided by Sagar M. Goyal at the University of Minnesota, MN, USA), rabbit anti-HA (Proteintech), and mouse anti-GAPDH (Proteintech).

### Peptide-conjugated morpholino oligomers

PPMOs were synthesized by covalently conjugating the peptide (RXR)_4_XB (where R is arginine, X is 6-aminohexanoic acid, and B is beta-alanine) to the 3′ end of the phosphorodiamidate morpholino oligomers (PMOs) through a non-cleavable linker, by methods described previously (46). The PMOs and peptide used to produce the PPMOs were purchased from Gene Tools LLC (Philomath, Oregon) and Peptide 2.0 (Chantilly, VA), respectively. The PPMO compounds were analyzed at the Mass Spectrometry Facility at Oregon State University, Corvallis, Oregon. All lyophilized PPMOs were resuspended in water to make a 1 mM stock solution.

### Cell culture experiments with PPMO

A549 cells were seeded in 6-well plates, unless specified, to be 70% confluent the following day. The cells were inoculated with HMPV at an MOI of 1.2 with synchronization by adding the virus to 700 µL of OptiMEM and incubating at 4°C for 1 h. Then, the cells were washed with PBS, 700 µL of fresh OptiMEM was added, and the cells were incubated at 37°C with 5% CO2. Unless specified, PPMOs were added at 1 hpi to achieve a concentration of 10 µM and incubated with cultures until the desired time point.

### Transfections

All transfections were performed as previously described (47). Briefly, 2 µg of DNA was mixed with 100 µL of OptiMEM and 4 µL of P3000 (Invitrogen). In a separate tube, 4 µL of Lipofectamine 3000 (Invitrogen) was mixed with 100 µL of OptiMEM. These two tubes were combined and incubated at room temperature for 15 min and then added dropwise to cell cultures. If a different amount of DNA was desired, DNA, OptiMEM, P3000, and Lipofectamine 3000 were scaled proportionally.

### Western blots

Cells were lysed using 0.15 M NaCl RIPA buffer containing protease inhibitors phenylmethanesulfonyl fluoride, iodoacetamide, aprotinin, and complete mini EDTA-free protease inhibitor tablets (Roche). A BCA assay was run for each lysate, and 10–15 µg of protein, diluted in SDS denaturing loading buffer and boiled, was loaded onto a 15% SDS-PAGE gel and separated at 60/100 V for the stacking and resolving layers, respectively. Proteins were then transferred to a 0.45 µM polyvinylidene difluoride (PVDF) membrane at 50 V for 2–3 h or overnight at 15 V. The membrane was blocked with 5% milk in tTBS (0.2% Tween-20 in Tris-buffered saline) for 1 h at 4°C and then incubated with primary antibodies (1:1,000 rabbit anti-M, 1:1,000 mouse anti-HMPV N, and 1:10,000 mouse anti-GAPDH) diluted in blocking buffer overnight at 4°C or for 3 h at room temperature. The anti-M antibody is a cross-reactive anti-avian metapneumovirus M antibody that also binds the HMPV M protein; hence, it was used in this study for immunoblotting. Membranes were washed with tTBS and incubated with secondary antibody from LICORbio (1:10,000 Goat anti-mouse 800 and 1:10,000 Goat anti-rabbit 680) diluted in blocking buffer for 1 h at room temperature. The membrane was then washed with tTBS and imaged on a Bio-Rad ChemiDoc and quantified by densitometry using Image Lab (Bio-Rad). Band intensities that were below the background signal were assigned an intensity level of 0. The data were quantified by dividing the GAPDH intensity of each sample by the maximum GAPDH signal on the western blot to obtain a normalization factor. Then, the intensity of the protein band of interest was divided by the normalization factor to obtain a GAPDH-normalized value. The GAPDH-normalized value of the control sample was averaged across the experimental replicates, and all other non-control samples were normalized to this averaged control value (48).

### Immunofluorescence

A549 cells were cultured on 10 mm coverslips and infected with WT HMPV. At each time point, the cells were washed with PBS and fixed with 4% paraformaldehyde (PFA) in PBS for 15 min at room temperature. Cells were then permeabilized with cold 1% Triton X-100 in PBS for 15 min at 4°C. Cells were washed with PBSN (PBS with 0.02% sodium azide) and then blocked with 1% normal goat serum (NGS) for 1 h at 4°C. When actin staining was needed, rhodamine phalloidin (Cytoskeleton) was added to the blocking step at a concentration of 10 nM. The coverslips were washed with PBSN and then incubated with primary antibodies (1:500 mouse anti-HMPV N, 1:500 mouse anti-HMPV F) overnight at 4°C or for 2 h at room temperature. Cells were washed with tPBSN (0.05% Tween 20 in PBSN) and incubated with secondary antibodies at room temperature for 1 h. The coverslips were mounted with Everbrite hard-set mounting medium with DAPI. Images were acquired using a spinning-disk Marianas confocal microscope system and analyzed using the Slidebook 6 software (Intelligent Imaging Innovations, Denver, CO) and Fiji/ImageJ (49).

### Fluorescence *in situ* hybridization

DNA probes used to stain for HMPV vRNA were prepared as previously described (45). Cellular samples used for FISH were fixed with 4% PFA for 15 min and permeabilized with 70% ethanol in water for at least 1 h at 4°C. Immunofluorescence staining (as previously described) was followed by fixation with 4% PFA for 15 min at room temperature. Cells were washed with Stellaris RNA FISH Wash Buffer A for 5 min at room temperature and then stained with DNA probes at a 1:100 dilution in Stellaris Hybridization Buffer overnight at 37°C. The following morning, the cells were washed with Stellaris RNA FISH Wash Buffer A for 30 min at 37°C and then stained with DAPI in Stellaris Wash Buffer A (0.16 µg/mL) for 30 min at 37°C. Cells were washed with Stellaris RNA FISH Wash Buffer B for 5 min at room temperature and mounted with Vectashield. Images were acquired with a spinning-disk Marianas system and analyzed using 3i Imaging Software. When IB properties were analyzed in Slidebook 6, cell boundaries were outlined, and signal thresholds were selected to exclude non-IB signals. Objects that had a major axis length smaller than 0.5 µm were omitted from IB analysis.

### Confocal microscopy and live-cell imaging

Fluorescent images were acquired using a spinning-disk Marianas system based on a Zeiss Axio Observer Z1 inverted fluorescence microscope with a Hamamatsu ORCA Fusion BT (sCMOS) camera. Images were acquired with a 100× Plan Apo PH NA 1.6 oil immersion objective, piezo stage controller, and spherical aberration correction module and controlled by the Slidebook 6 software (Intelligent Imaging Innovations, Denver, CO). Acquired Z-stacks have z-steps between 250 and 300 nm. An Ibidi four-well chamber slide was used for live-cell imaging in a temperature-controlled chamber set to 37°C. Unless specified, live-cell imaging occurred between 24 and 26 hpi and was acquired at approximately every 0.5–1 s intervals. To increase acquisition speed, only single z-slices were acquired for each time-lapse. Track speeds were determined by monitoring mCherryP movements in treated cells every 0.5 s with the Manual Particle Tracking software in 3i Slidebook 6. This measured the track speed between each time interval, which was used to calculate the average and maximum track speeds.

### Flow cytometry

A549 cells were washed with cold PBS and blocked with 5% bovine serum albumin (BSA) for 30 mi at 4°C. Cells were then incubated with primary antibody (1:500 mouse anti-HMPV F) for 1 h at 4°C. Cells were washed with 1% BSA and then incubated with secondary antibody Jackson ImmunoResearch (1:500 Goat anti-mouse 488) for 1 h at 4°C and washed with PBS. Cells were lysed with 10 mM EDTA in PBS for 5 min at room temperature and then centrifuged at 1,150 × *g* for 2 m. The supernatant was aspirated, and the cells were resuspended in 1% PFA and incubated for 30 min at 4°C and then injected into the Millipore Guava EasyCyte HT. In experiments with rgHMPV, cells were immediately lifted, fixed, and loaded onto the Millipore Guava EasyCyte HT with no antibody staining. Cells were gated by forward and side scatter parameters, and a total of 10,000 cells were counted, with infected cells gated by fluorescence.

### RNA extraction and quantitative PCR

Duplicate samples of A549 cells were infected with WT HMPV. At each time point, cells were washed with PBS, and RNA was extracted using the Bio-Rad Aurum Total RNA Mini Kit as stated by the manufacturer’s procedures. One-step RT-qPCR was performed using the Bio-Rad iTaq Universal SYBR Green One-Step Kit. Briefly, a 25 µL reaction containing 70 ng of RNA, 1 µM of each FW and RV primer targeting the N, M, or F genes, 1× SYBR Green Reaction Mix, and 0.25 µL of reverse transcriptase was used. Cycle parameters were 10 min at 50°C, 5 min at 95°C, followed by 40 cycles of 10 s at 95°C and 30 s at 60°C. For two-step qPCR, reverse transcription using Promega AMV Reverse Transcriptase was performed as previously described (45). Briefly, cDNA was synthesized by using 500 ng of RNA, 1.25 mM dNTPs, 5 U of AMV Reverse Transcriptase, 1× reaction buffer, and 1.25 mM of either oligo(dT)_20_ primer (for mRNA amplification) or vRNA primer 5′-AACGCGTATAAATTAAGTTAC-3′ (for vRNA amplification). The reaction was incubated for 10 min at 80°C and then 1 h at 42°C. PowerTrack SYBR Green Master Mix was used with 2 µL of cDNA, 0.5 µM of both FW and RV primers targeting the N, M, or F genes, and run for 2 min at 95°C, followed by 40 cycles of 15 s at 95°C and 1 min at 60°C. Both one- and two-step experiments were run on a Bio-Rad CFX Connect Real-Time System and analyzed on CFX Maestro Software and Microsoft Excel. If a reaction did not reach the signal threshold after 40 cycles, the Cq value was set as the total number of cycles + 1. Total vRNA, vmRNA, and vgRNA were quantified by subtracting the target RNA C_q_ from Actinβ C_q_ (ΔC_q_) and averaging technical duplicate ΔC_q_ values. The experimental replicate ΔC_q_ values for PPMO_NC_ were averaged, and each experimental ΔC_q_ value was subtracted from the averaged PPMO_NC_ ΔC_q_ (ΔΔC_q_) and then using the ΔΔC_q_ as shown: 2^−ΔΔC_q_^ and normalize that value to PPMO_NC_-treated infection as a percent.

## RESULTS

### PPMO design

**Fig 1:**
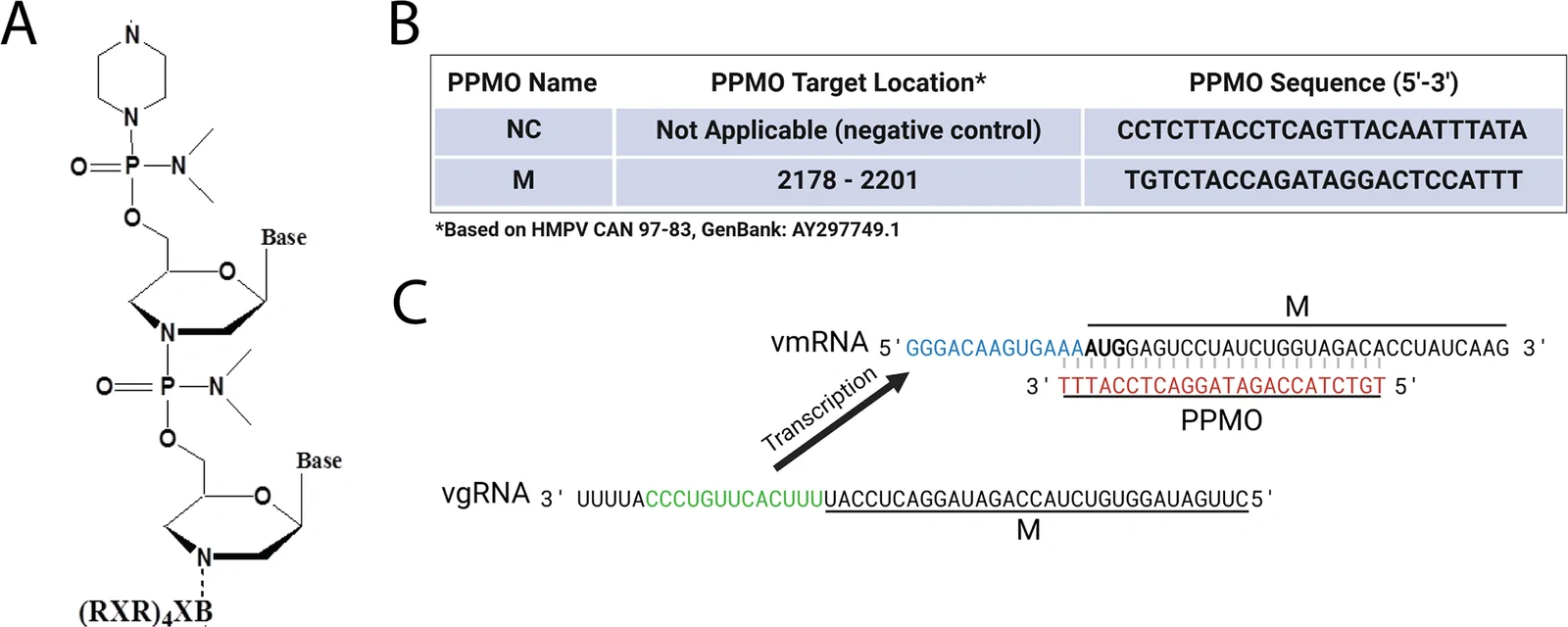
PPMO design. (**A**) Schematic structure of a peptide-conjugated phosphorodiamidate morpholino oligomer. Illustrated are two representative PMO subunits, each containing a morpholine ring and nucleobase, with the subunits connected by phosphorodiamidate linkages. The PMO is conjugated to a single cell-penetrating peptide (CPP) to produce PPMO. For this study, the CPP was (RXR)_4_XB (sometimes abbreviated “P7”), where R is arginine, X is 6-aminohexanoic acid, and B is beta-alanine, and was conjugated at the 3′ end of each PMO. (**B**) Table of the main PPMOs used in this study and their respective targets and sequences. The location of the PPMO_M_ target sequence is shown based on the HMPV CAN 97-83 genome (Accession number AY297749.1). (**C**) The alignment of PPMO_M_ on the target viral mRNA (vmRNA) sequence. Green bases represent the gene start (GS) sequence, and the blue bases show its complement; bolded bases are the initiating codon, and red bases are the PPMO. The M gene ORF sequence is indicated in both the vmRNA and viral genomic RNA (vgRNA).

The M protein has been shown to interact with several viral proteins, suggesting that it may have other roles beyond virus assembly and budding (23, 33, 50). In this investigation of the HMPV M protein, we utilized an antisense PPMO designed to block translation of M mRNA. Phosphorodiamidate morpholino oligomers (PMOs), also termed “morpholinos,” are single-stranded-nucleic acid analogs containing the same nucleobases as DNA but linked through a non-natural backbone (51) (Fig. 1A), conferring stability against nucleases. PMOs typically comprise 20–25 nucleobase-containing subunits and exert their antisense effect through steric blockade. PMOs hybridize to single-stranded RNA in a sequence-specific manner and can thereby interfere with the normal functions of targeted RNA. The PMO:RNA duplex does not form a substrate for RNase H, thus increasing target specificity (52, 53). To facilitate cellular entry, a PMO can be conjugated to a cell-penetrating peptide (CPP) to produce a PPMO (54–57). PPMOs are water-soluble, nuclease-resistant, and non-toxic at effective concentrations across a range of *in vitro* and *in vivo* applications (58–60). PPMOs targeting viral sequences have suppressed the growth of numerous RNA and DNA viruses in cell cultures and animal models by reducing viral RNA translation, disrupting viral RNA secondary structures in untranslated regions (UTRs), or by interfering with *cis*-acting RNA:RNA interactions known to have important roles in virus replication (56, 60–64).

For this study, two PPMOs were designed: one PPMO targeting HMPV M mRNA (PPMO_M_) and a non-specific negative control PPMO (PPMO_NC_) (Fig. 1B). PPMO_NC_ contains a non-targeting PMO sequence that has little homology with any transcript from an RNA virus, human, or mouse, as determined by BLASTn, and is used to control for cytotoxic or off-target effects caused by PPMO compounds. When designing PPMO_M_, we focused on the sequence surrounding the AUG start codon of HMPV M mRNA. This region has historically been a productive region to target for reducing the expression of specific proteins. However, the 5′ UTRs of the mRNAs for several different HMPV genes are conserved in the region corresponding to nucleotide positions −13 to −1 relative to the start codon (65) (Fig. S1). To avoid this highly conserved sequence, we instead targeted bases −2 to +22 (relative to the A of the initiating AUG start codon) of M mRNA (Fig. 1C) to specifically target HMPV M. PPMO_M_ (red) targets the HMPV M mRNA-coding sequence and has little complement with the M gene 5′ UTR sequence (blue).

### PPMOs reduce M in infection and alter N localization

**Fig 2:**
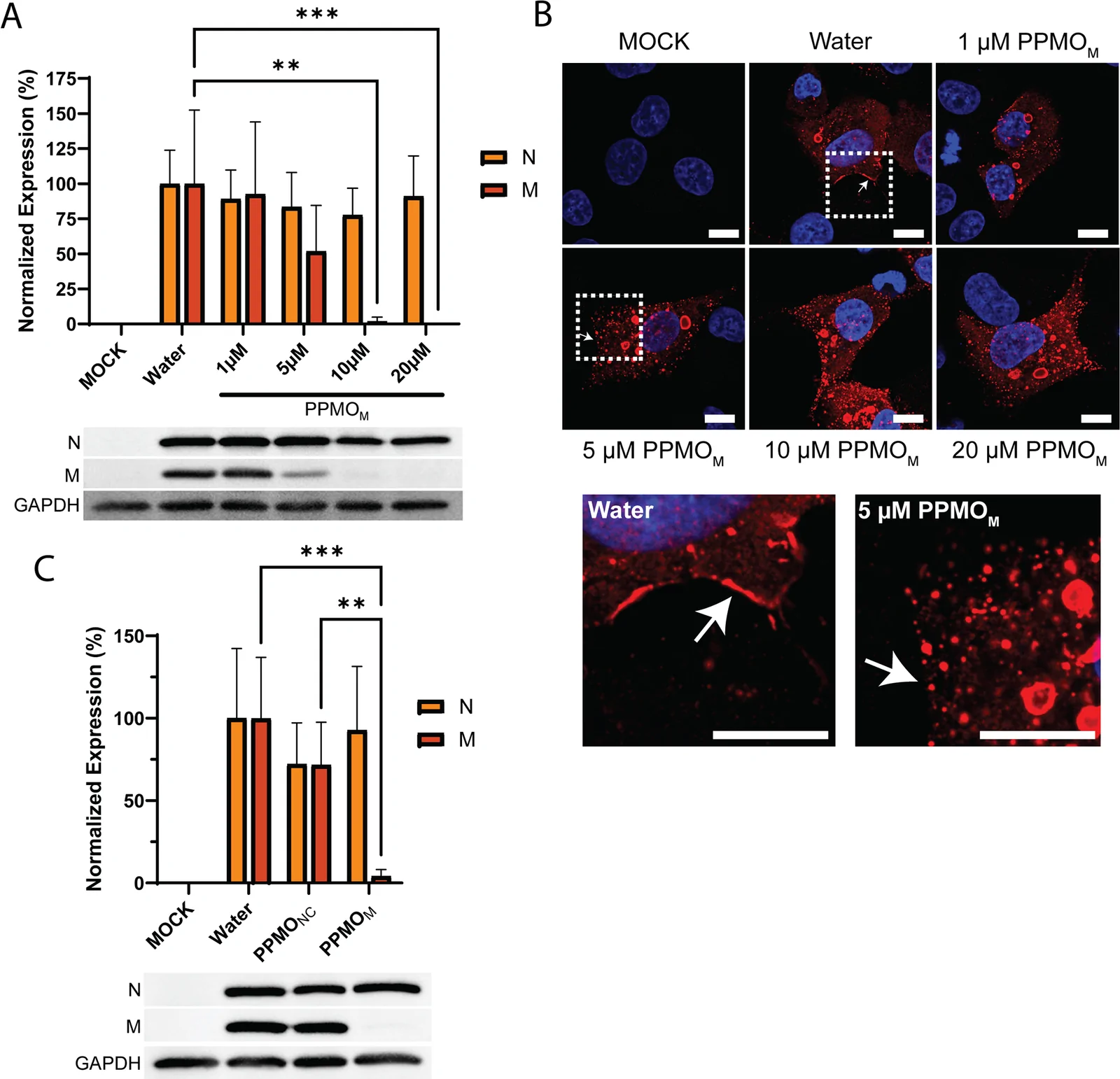
PPMO_M_ treatment specifically reduces M protein expression and alters N protein localization in infected cells. HMPV-infected or MOCK-infected A549 cells were treated with water or varying concentrations of PPMO_M_ at 1 hpi. (**A**) At 24 hpi, the cells were lysed and used for immunoblotting with anti-GAPDH, anti-M, and anti-HMPV N antibodies. Data are from three independent experiments and shown as the mean ± SD (*n* = 3). (**B**) At 24 hpi, the cells were fixed and stained with an anti-HMPV N (red) antibody and DAPI (blue) and examined by confocal microscopy. Data are from three independent experiments, and a single representative experiment is shown as a single z-slice. The magnified regions are outlined by dashed white boxes, and the white arrows point to the edge of the cell. Scale bars are 10 µm. (**C**) A549 cells were MOCK-infected or infected with WT HMPV and treated with water, 10 µM PPMO_NC_, or 10 µM PPMO_M_ at 1 hpi. The infection was allowed to progress until 24 hpi, and then the cells were lysed and used for immunoblotting with anti-GAPDH, anti-M, and anti-HMPV N antibodies. Data are from four independent experiments and shown as the mean ± SD (*n* = 4). **P < 0.01, ***P < 0.001, by two-way ANOVA.

PPMOs have been previously used to decrease the expression of viral proteins (39, 41, 60), particularly as a potential means of inhibiting infection. Prior work with PPMOs has primarily focused on monitoring viral titers, and several studies have utilized varying levels of PPMOs to assess the level needed to inhibit viral propagation (40, 44, 61, 64, 66). In order to help characterize the role of the M protein in infected cells, we instead tested if PPMO_M_ treatment could dose-dependently decrease M protein levels. A549 cells infected with wild-type (WT) HMPV CAN 97-83 were treated with the vehicle (water) or varying concentrations of PPMO_M_ at 1 hour post-infection (hpi). At 24 hpi, the cells were lysed and examined for protein expression. The M protein levels were dose-dependently reduced by 7.4% (1 µM), 48.2% (5 µM), 97.9% (10 µM), and >99% (20 µM) when compared to vehicle treatment (Fig. 2A). The HMPV N protein was also examined to verify that PPMO_M_ did not non-specifically inhibit the expression of other viral genes. The N protein levels were only modestly reduced, by 10.8% (1 µM), 16.4% (5 µM), 22.3% (10 µM), and 8.9% (20 µM) (Fig. 2A), indicating that the reductions in M protein expression were specific. No cell cytotoxicity was observed at 10 µM concentrations (Fig. S2). Since 10 µM PPMO_M_ treatment resulted in a >95% decrease in M protein expression and had no cytotoxic effects, a 10 µM treatment concentration was used for subsequent experiments when a near-total reduction in M protein expression was desired.

Since M protein expression could be controlled during infection using PPMOs, we examined the effect of increasing PPMO_M_ on HMPV-infected cells, with HMPV N protein localization as one of the markers. Immunofluorescence microscopy was used to monitor N localization at varying PPMO_M_ concentrations. Interestingly, a 1 µM treatment of PPMO_M_ applied at 1 hpi did not observably alter infection characteristics, but a 5 µM or higher concentration decreased filamentous-N on the cell boundary, likely correlating with a loss of the N protein in viral filaments (Fig. 2B). Infections treated with 5 µM PPMOs had reduced filamentous-N, but N was still present on the periphery of some cells, while 10 and 20 µM treatments had even less filamentous-N. This suggests that when M protein levels are reduced by approximately 50% or more, there are significant changes in virus assembly, filament maturation, and/or trafficking. PPMO_M_ treatments that resulted in approximately a 50% or greater reduction in the M protein had an increased number of IBs when compared to the water-treated control (Fig. 2B). This could be because the M protein was not expressed to a level needed to efficiently facilitate trafficking of vRNPs to assembly sites, leaving vRNPs within IBs throughout the cell. These experiments verify that the levels of PPMO_M_ needed to significantly reduce M protein levels match the levels of PPMO_M_ needed to see observable differences in infected cells.

We also used PPMO_NC_ as a control to test for any generic, non-specific impacts of PPMOs on infection. Cells were treated with 10 µM PPMO_NC_ or PPMO_M_, and N and M expression was quantified. Infections treated with PPMO_M_ reduced M protein expression by 95%, and no significant change in N expression was observed (Fig. 2C). However, a modest decrease in the M (28.3%) and N (27.8%) protein expression was also observed in PPMO_NC_-treated infections compared to the vehicle control (Fig. 2C). Further experiments were performed to determine the cause of this apparently non-specific effect. We found a minor decrease in HMPV infection rate when cells were treated with any PPMO at 1 hpi, regardless of the PMO nucleobase sequence (Fig. S3). This reduction in infection was not observed when cells were treated with PPMO at 6 hpi (Fig. S4). We hypothesize that this non-specific effect on infection rate is due to interference with virus entry into cells, as the effect is not present when infected cultures were treated at a later time. Considering that the non-specific effect of PPMO_NC_ was relatively minor in relation to the specific reduction of M expression with the M-targeted PPMO, we utilized PPMO_NC_ as a control in all subsequent experiments.

### Viral RNA localization changes with the loss of M

**Fig 3:**
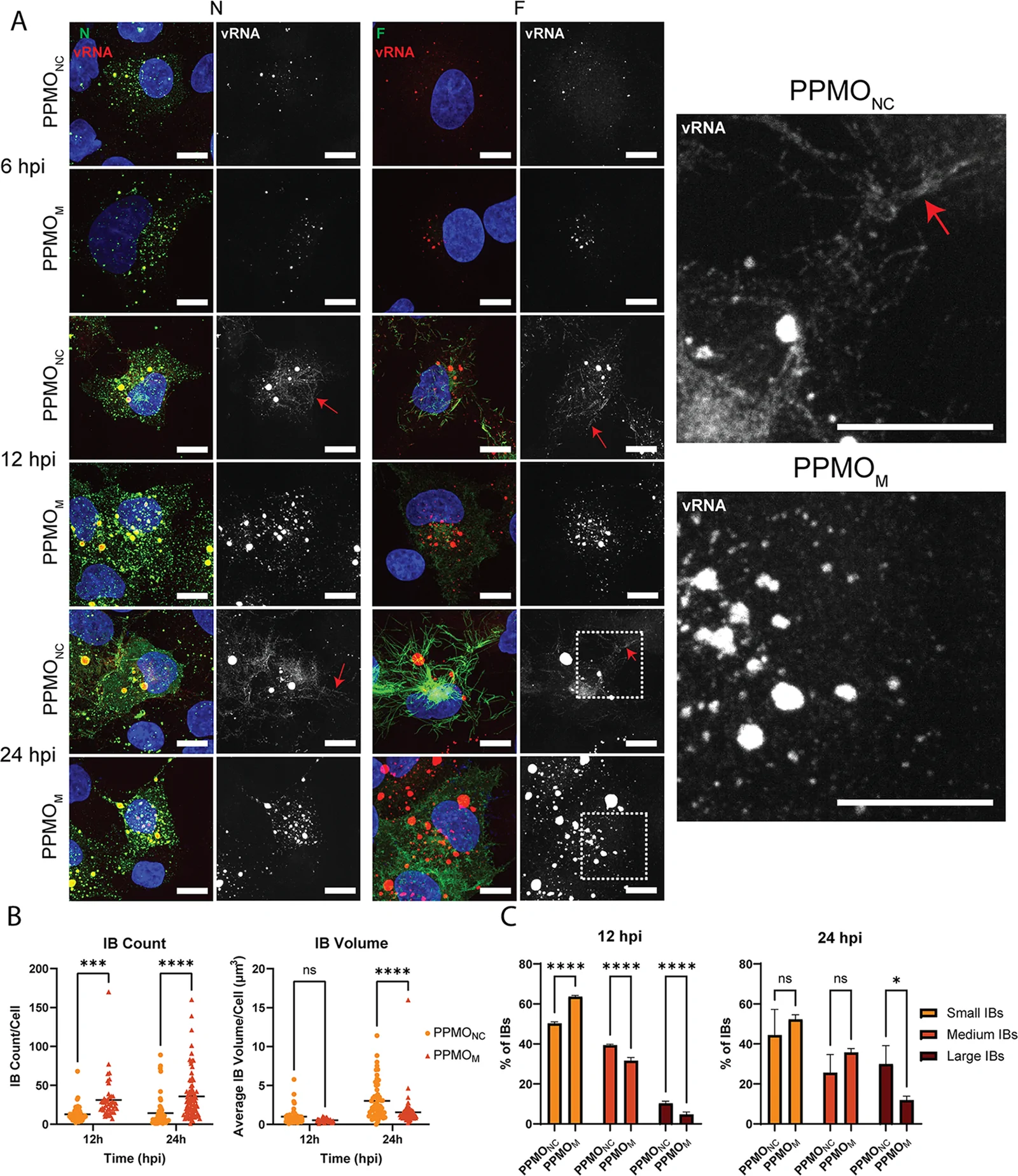
Viral RNA localization affected by the presence of M protein. A549 cells were infected with WT HMPV and treated with 10 µM PPMO_NC_ or PPMO_M_ at 1 hpi. At 6, 12, or 24 hpi, (**A**) the cells were fixed and stained with anti-HMPV N (green; left) or anti-HMPV F (green; right) antibodies. FISH staining for vRNA (red/white) was then performed, followed by DAPI (blue) staining. Samples were imaged by confocal microscopy and are shown as maximum intensity projections. Data are from three independent experiments, and a single representative experiment is shown. Red arrows show vRNA filaments, and the magnified region is shown by a white box. Scale bar is 10 µm. (**B**) IB count and volume were quantified (per cell) using vRNA as a marker for IBs. The images were then analyzed to detect individual IBs and their volumes per cell. At least 45 cells from each condition were analyzed across three independent experiments and plotted individually with the mean (black line). (**C**) IBs were grouped into three sizes: small (<0.2 µm^3^), medium (between 0.2 and 3 µm^3^), and large (>3 µm^3^). The percentage of IBs was calculated by dividing the number of IBs within a group by the total number of IBs in that cell. Each independent experiment contained at least 10 cells, which were averaged and represented as a single data point. Data are represented as the average of three independent experiments and shown as the mean ± SD (*n* = 3). ns, not significant, *P < 0.05, ***P < 0.001, ****P < 0.0001, by two-way ANOVA.

As demonstrated above, N protein localization was affected by the level of M protein present (Fig. 2B), suggesting a role for the HMPV M protein in the localization of vRNPs. To further characterize this potential role, FISH probes were used to detect HMPV vRNA at different times post-infection in cells infected with HMPV and treated with 10 µM PPMOs at 1 hpi. At 6, 12, and 24 hpi, the cells were fixed and stained for vRNA and the viral N and F proteins. At 6 hpi, HMPV vRNA is localized in IBs and colocalizes with the HMPV N protein (Fig. 3A). No significant difference in vRNA distribution was observed at 6 hpi for PPMO_NC_ and PPMO_M_-treated infections (Fig. 3A). At 12 and 24 hpi, the PPMO_NC_-treated infections had linear vRNA staining patterns, shown by red arrows, likely indicating vRNA in mature viral filaments (Fig. 3A). The PPMO_M_-treated infections showed a very different staining pattern at 12 and 24 hpi, with few vRNA filaments observed and an increase in the number of IBs (Fig. 3A). This shows that reductions in M protein levels affect vRNA localization in infected cells at the later stages of infection. HMPV F staining was also examined. In PPMO_NC_-treated cultures, mature F-stained filaments were observed, which present as long protrusions extending from the cell membrane (Fig. 3A). In PPMO_M_-treated cultures, only short viral filaments extending from the cell body were observed, which are distinctly different in appearance from the traditional mature filaments found in normal infections (Fig. 3A). We termed these shorter structures immature filaments because they are not elongated like typical HMPV filaments. In the PPMO_NC_ control, vRNA filaments colocalized with mature HMPV F filaments, while cells receiving PPMO_M_ treatment lacked vRNA signal along the periphery of infected cells. This suggested a potential role for the M protein in trafficking vRNA to assembly sites and producing mature viral filaments.

To quantify this change in vRNA localization, we utilized FISH-stained samples to calculate the average IB count and volume per cell. PPMO_M_ treatment increased IB count per cell from 12.7 to 31.4 at 12 hpi and from 14.2 to 35.8 at 24 hpi when compared to PPMO_NC_ (Fig. 3B). Additionally, the average IB volume per cell decreased from 0.99 µm^3^ to 0.54 µm^3^ at 12 hpi and from 3.02 µm^3^ to 1.56 µm^3^ at 24 hpi when the M protein was knocked down (Fig. 3B). Taken together, these findings suggest a role for the M protein in IB properties, potentially due to changes in vRNA trafficking.

To further refine this analysis, IB sizes were divided into three groups: small (<0.2 µm^3^), medium (0.2–3 µm^3^), and large (>3 µm^3^). When the M protein was reduced, there was an increase in the percentage of small IBs and a decrease in medium- and large-sized structures at 12 hpi (Fig. 3C). At 24 hpi, the percentage of small and medium IBs showed no significant change, whereas a significant decrease in large IBs was observed (Fig. 3C). This suggests that the M protein plays a role in IB dynamics. Altogether, these results indicate that the HMPV M protein plays a pivotal role in vRNA localization.

### M does not impact viral transcription, replication, or N-P interactions

**Fig 4:**
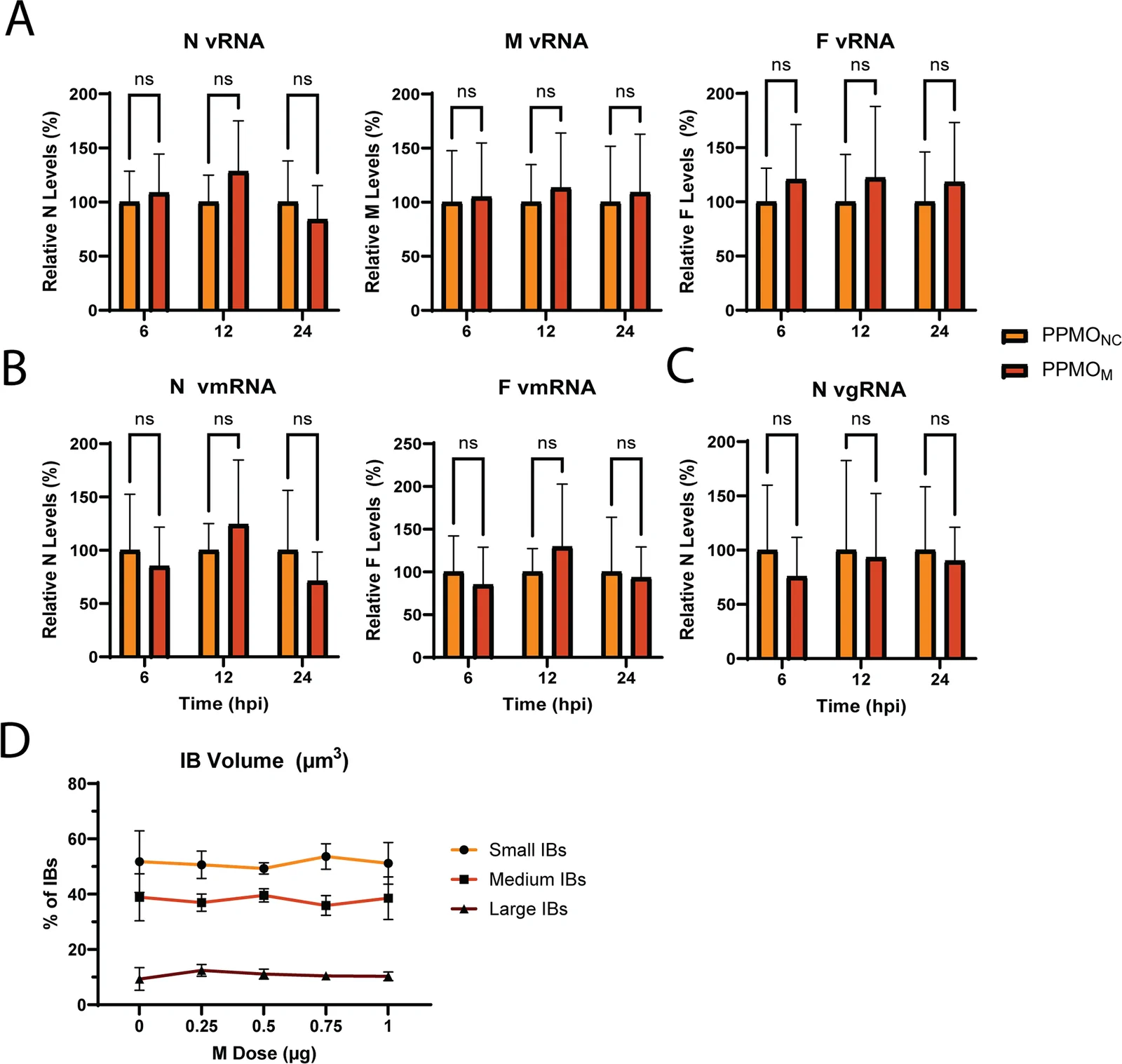
The M protein does not impact viral transcription or replication. A549 cells were MOCK-infected or infected with WT HMPV and subsequently treated with water, 10 µM PPMO_NC_, or 10 µM PPMO_M_ at 1 hpi. At 6, 12, or 24 hpi, the cells were lysed, and RNA was extracted and purified. (**A**) Total RNA was quantified using one-step RT-qPCR using primers targeting Actinβ, HMPV N, M, and F. (**B**) Viral mRNA (vmRNA) was quantified by running cDNA synthesis and then qPCR for Actinβ, HMPV N, and HMPV F. (**C**) Viral genomic RNA (vgRNA) was quantified with cDNA synthesis and then qPCR for HMPV N. Data are from three independent experiments, each in duplicate, and shown as the mean of the experimental replicates ± SD (*n* = 3). (**D**) A549 cells were plated and transfected with codon-optimized pCAGGS-HMPV N, pCAGGS-HMPV mCherryP, and varying doses of pCAGGS-HMPV HA-M (69); 24 h post-transfection, the cells were fixed and imaged through confocal microscopy. IB-like structures were thresholded by the mCherry signal, and their volume was measured. Objects below a major axis length of 0.5 µm were excluded from analysis. IB-like structures were grouped into three sizes: small (<0.2 µm^3^), medium (between 0.2 and 3 µm^3^), and large (>3 µm^3^). The percentage was calculated by dividing the number of IB-like structures within a group by the total number in that cell, and then averaging this value across all of the cells in each treatment. A total of at least 80 cells were measured across three experimental replicates, and the average of each IB size group/cell was calculated as a percent. ns, not significant by two-way ANOVA.

Changes in IB size when the M protein was reduced could result from changes in viral replication and transcription. Previous research showed that the M proteins of some NNSVs impact viral replication and transcription (25, 67, 68). To test whether viral RNA synthesis is impacted by M expression, we utilized one-step qPCR to evaluate all vRNA synthesis products from replication and transcription by quantifying N gene abundance. Surprisingly, there were no significant changes in total N vRNA at 6, 12, or 24 hpi (Fig. 4A). To test whether the other genes were similar to N, HMPV M, and F were also investigated. Both genes showed no significant change in vRNA abundance at 6, 12, and 24 hpi when the level of M was reduced (Fig. 4A). Together, these results suggest that reduced HMPV M protein expression does not significantly impact total viral RNA synthesis.

To assess the impact of the HMPV M protein on replication or transcription separately, two-step qPCR was utilized to examine viral messenger (vmRNA) or genomic (vgRNA) RNA. HMPV N and F mRNA synthesis was examined because they are the first and fourth genes in the genome, respectively; hence, any changes in N or F transcripts would indicate changes in transcription or polymerase termination. The qPCR results showed no significant change in N or F vmRNA at any time point, suggesting that the M protein does not significantly alter viral transcription (Fig. 4B). vgRNA was also analyzed to evaluate changes in antigenome-to-genome synthesis. The results showed that vgRNA had no changes in abundance relative to the PPMO_NC_-treated control cells at any time point (Fig. 4C), implying that M does not affect viral transcription or replication.

Since vRNA abundance does not change when M protein expression is reduced, it is possible that M disrupts the protein-protein interactions needed for IB formation. Given that RSV M interacts with P (23), we investigated whether the HMPV M protein interfered with the HMPV N and P interactions. A549 cells were transfected with HMPV N, mCherry-tagged P (mCherryP), and varying doses of HA-tagged M (HA-M). At 24 hpt, the cells were fixed and imaged to detect the IB-like structures formed by the transient expression of N and P. The IB-like structure sizes were then sorted into three groups: small (<0.2 µm^3^), medium (0.2–3 µm^3^), and large (>3 µm^3^). Quantification of these structures showed no significant change in IB size distribution in the absence of the M protein or when more M was added into the transfections (Fig. 4D). Together, this suggests that the M protein does not play a direct role in producing larger IBs but rather modulates other aspects of infection that affect IB size.

### The M protein is involved in ribonucleoprotein localization

**Fig 5:**
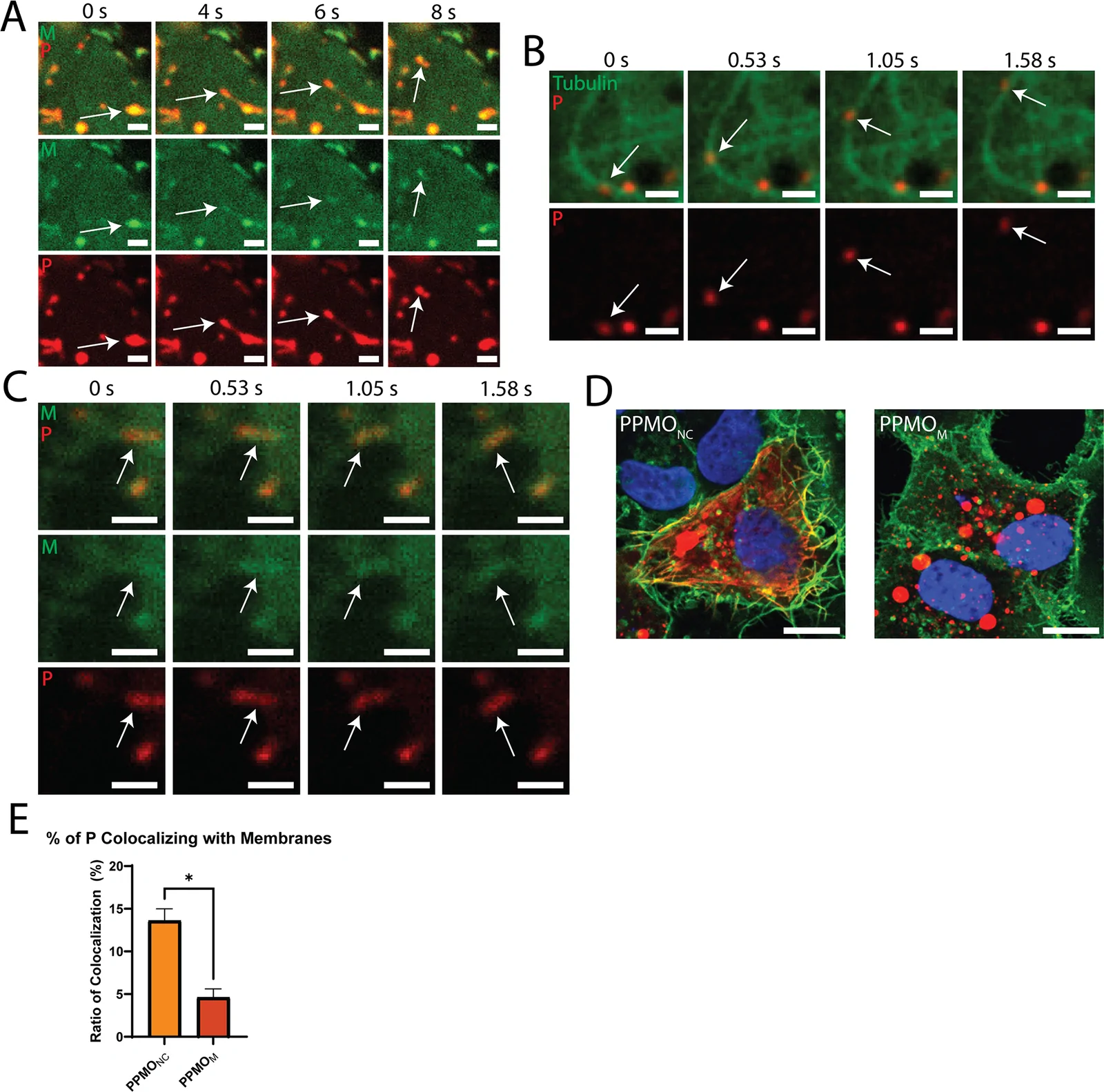
The M protein is involved in ribonucleoprotein localization. (**A**) A549 cells were infected with WT HMPV for 3 h, washed, and subsequently transfected with 1 µg GFP-M (green) and 1 µg mCherryP (red) at 3 hpi. At 24 hpi, live cells were imaged. Shown is a representative single z-slice time-lapse acquired every 1 s, and the selected frames are shown. The white arrows show a vRNP complex moving throughout the cell. (**B**) mCherryP (red) was observed trafficking along eGFP-tagged α-tubulin (Addgene Plasmid #21039) (green) (70). Scale bar is 1 µm. (**C**) A549 cells were infected with WT HMPV for 3 h, washed, and subsequently transfected with 1 µg GFP-M (green) and 1 µg mCherryP (red) at 3 hpi. At 24 hpi, live cells were imaged. Scale bar is 1 µm. (**D**) A549 cells were plated and infected with WT HMPV for 3 h, washed, and subsequently transfected with 2 µg of pCAGGs-mCherryP (red) at 3 hpi. At 6 hpi, 10 µM of PPMOs were added. At 24 hpi, the cells were fixed and stained with Alexa Fluor 647-conjugated WGA (pseudo-colored green) and DAPI (blue) and imaged by confocal microscopy. A single z-slice is shown, and the scale bar is 10 µm. (**E**) At least 10 cells were measured in each replicate, which were averaged and represented as a single data point, and analyzed to measure the colocalization of mCherryP and WGA. Data are represented as the average of three independent experiments and shown as the mean ± SD (*n* = 3). *P < 0.05, by a paired *t* test.

As we have shown, HMPV M does not impact vRNA synthesis or protein-protein interactions that lead to IB formation. Therefore, we hypothesized that the M protein plays a role in trafficking vRNPs. To test this, mCherryP was used to track vRNPs in live cells. A549 cells were infected with WT HMPV and subsequently transfected with mCherryP and an M protein that is N-terminally tagged with GFP (GFP-M) at 3 hpi. At 24 hpi, the infected and transfected cultures were imaged. Interestingly, we observed colocalization of the GFP-M and mCherryP proteins in IBs (Fig. 5A), like in RSV (22).

The GFP-M and mCherryP proteins were also observed moving together in what appeared to be direct linear motions that originated from IBs (white arrow), suggesting that M traffics with vRNPs (Fig. 5A). Based on previous literature for NNSVs, we hypothesized that vRNP movement was microtubule-dependent (71–73). Therefore, we investigated HMPV vRNP movement along microtubules. Our results showed that vRNPs do traffic along microtubules (Fig. 5B). We also observed some colocalization of mCherryP and Rab11a, but the interaction was more evident when the proteins moved together in live cells (Fig. S5), supporting a potential role for Rab11^+^ recycling endosomes in *pneumoviruses* (71).

We then focused on looking at vRNP movement into the peripheral cytoplasm of infected cells. Using live-cell imaging with the mCherryP and GFP-M proteins, we observed some M-vRNPs move into structures resembling immature filaments (Fig. 5C). This shows the M-vRNPs can eventually localize in assembly sites. We then analyzed mCherryP localization at the plasma membrane under PPMO treatment. A549 cells were infected with WT HMPV and subsequently transfected with mCherryP at 3 hpi. At 6 hpi, the cells were treated with 10 µM PPMOs, fixed at 24 hpi, and stained with wheatgerm agglutinin (WGA) to label the membranes. PPMO_NC_-treated cells showed clear localization of the mCherryP protein at the inner surface of the plasma membrane (Fig. 5D). In contrast, PPMO_M_-treated infections showed significantly less mCherryP localization at membranes (Fig. 5D). This showed that in the absence of M protein, the P protein is not retained on the plasma membrane. To quantify this difference, WGA and P signal were thresholded, and the amount of colocalization was derived by dividing the area of WGA-P colocalization by the total WGA area and expressed as a percentage. In some instances, cells expressed the mCherryP protein at very high or low levels, leading to over- or under-thresholding of the signal, respectively. To overcome this challenge, cells were thresholded for mCherryP signal, and the mCherryP area was divided by the total area of each cell and expressed as a percentage. If this ratio was below 0.5% or above 5%, then the cell was not included in the analysis. The quantification of WGA and P colocalization showed a significant decrease in mCherryP signal at membranes when the M protein was reduced (Fig. 5E). The colocalization decreased from 13.6% with PPMO_NC_ to 4.6% with PPMO_M_, suggesting that the M protein plays a role in mCherryP protein localization to the plasma membrane. This work supports our hypothesis that the HMPV M protein is involved in vRNP localization and likely promotes the association of vRNPs and various viral proteins to immature assembly sites.

### The M protein is not necessary for viral RNP movement

**Fig 6:**
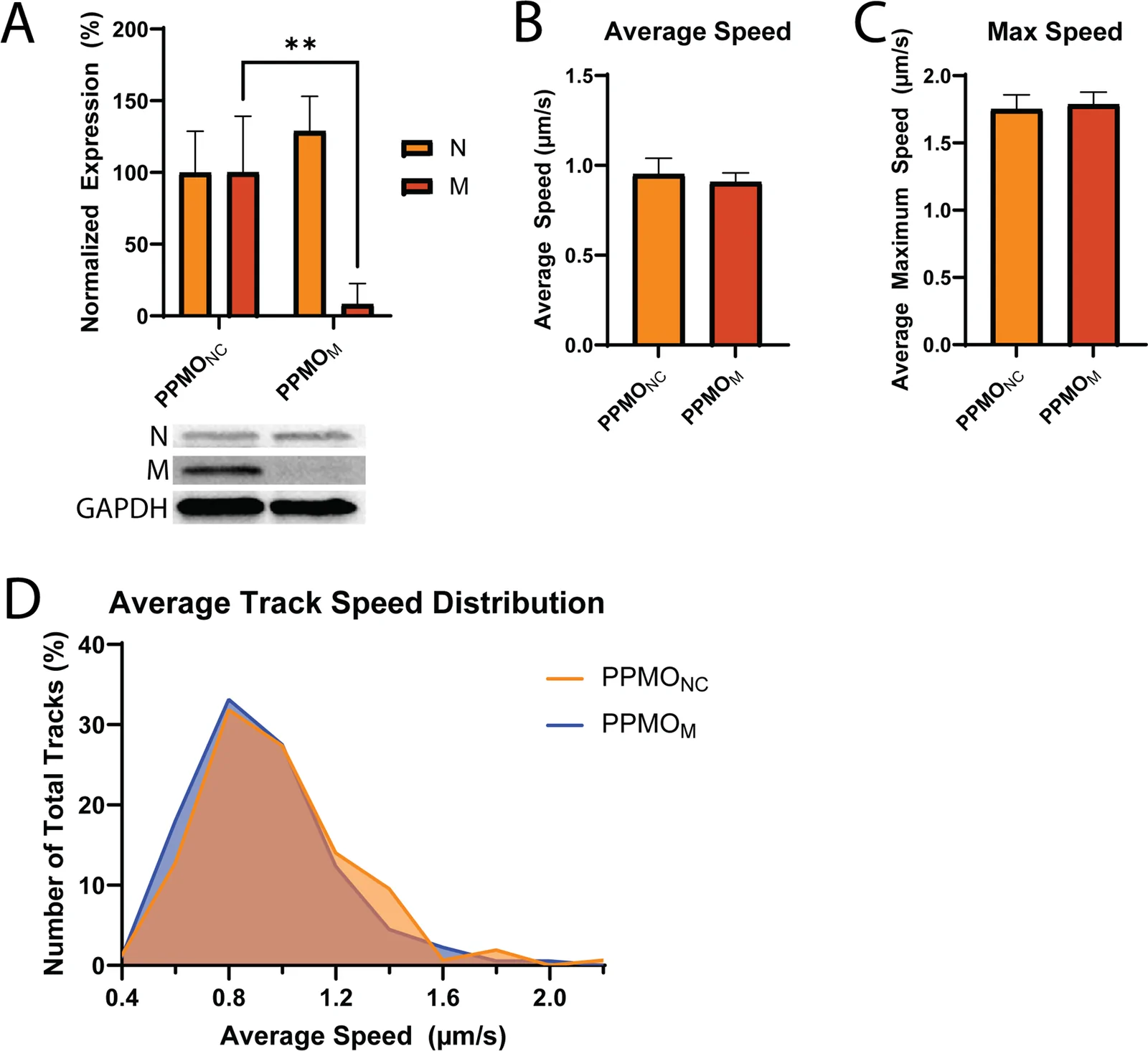
The M protein is not necessary for viral RNP movement. A549 cells were infected with WT HMPV for 3 h, washed, and transfected with mCherryP at 3 hpi. At 6 hpi, the media were changed, and 15 µM PPMOs were added. At 24 hpi, the cells were lysed and analyzed with immunoblots with (**A**) anti-M, anti-N, and anti-GAPDH antibodies. Before lysis, infected cells were imaged in real time to track mCherryP signal at approximately 500-ms intervals for 1 min. Tracks were manually measured from start to stop. The (**B**) average and (**C**) average maximum speeds for at least 150 tracks were analyzed for each treatment condition. Three independent experiments are shown as the mean ± SD (*n* = 3). (**D**) The average speed of each track measured is shown in a histogram where the tracks are grouped into bins of 0.2 µm/s. **P < 0.01, by two-way ANOVA.

We have shown that the HMPV M protein traffics with vRNP complexes to the cell periphery (Fig. 5C). Therefore, we hypothesized that the M protein may be responsible for associating vRNP complexes with endosomes. However, previous studies indicated that the *paramyxoviridae* and *pneumoviridae* C and L proteins associate vRNPs with Rab11a^+^ endosomes (71, 74, 75). To test whether this association between vRNPs and endosomes is M-dependent for HMPV, A549 cells were infected with WT HMPV and transfected with mCherryP at 3 hpi. At 6 hpi, the co-infection/transfection was treated with 15 µM PPMOs. A 15 µM treatment was chosen because both infection and transfection may reduce PPMO entry into cells. In cells treated with PPMO_M_, the HMPV M levels decreased to 8.3%, and N protein expression was slightly increased to 128.9% of that observed in cells treated with PPMO_NC_ (Fig. 6A). The elevated N levels observed may be due to decreased budding, resulting in more viral proteins being retained in cells.

After confirming that M protein expression was reduced in co-infection/transfected cells, vRNPs were manually tracked with live cell imaging. Viral RNP movement properties varied, with some moving more quickly than others and some tracks being shorter or longer. At least 150 tracks were measured from three independent experiments and showed no difference in the average speed of vRNPs between PPMO_NC_ and PPMO_M_ treatments (Fig. 6B). The average speed of these tracks was 0.95 and 0.91 µm/s for PPMO_NC_ and PPMO_M_ treatments, respectively. The average maximum speed per track was also similar between the treatments at 1.75 and 1.78 µm/s for PPMO_NC_- and PPMO_M_-treated infections, respectively (Fig. 6C). Both PPMO treatments also had a similar number of tracks in a single z-plane, averaging 7.5 and 6.9 tracks/cell/min for PPMO_NC_ and PPMO_M_, respectively. To better visualize these results, a histogram of individual vRNP track speeds was plotted (Fig. 6D). Both PPMO_NC_ and PPMO_M_ had a similar distribution of slow- and fast-moving vRNPs, suggesting that there were no changes in viral vRNP movements when the M protein was reduced (Fig. 6D). This suggests that vRNP movements along microtubules do not depend on the M protein but that M plays a role in vRNP localization to the plasma membrane later in infection. Therefore, the M protein may function in the later stages of trafficking to retain vRNPs at the cell periphery.

### HMPV M protein levels directly correlate with viral particle production

**Fig 7:**
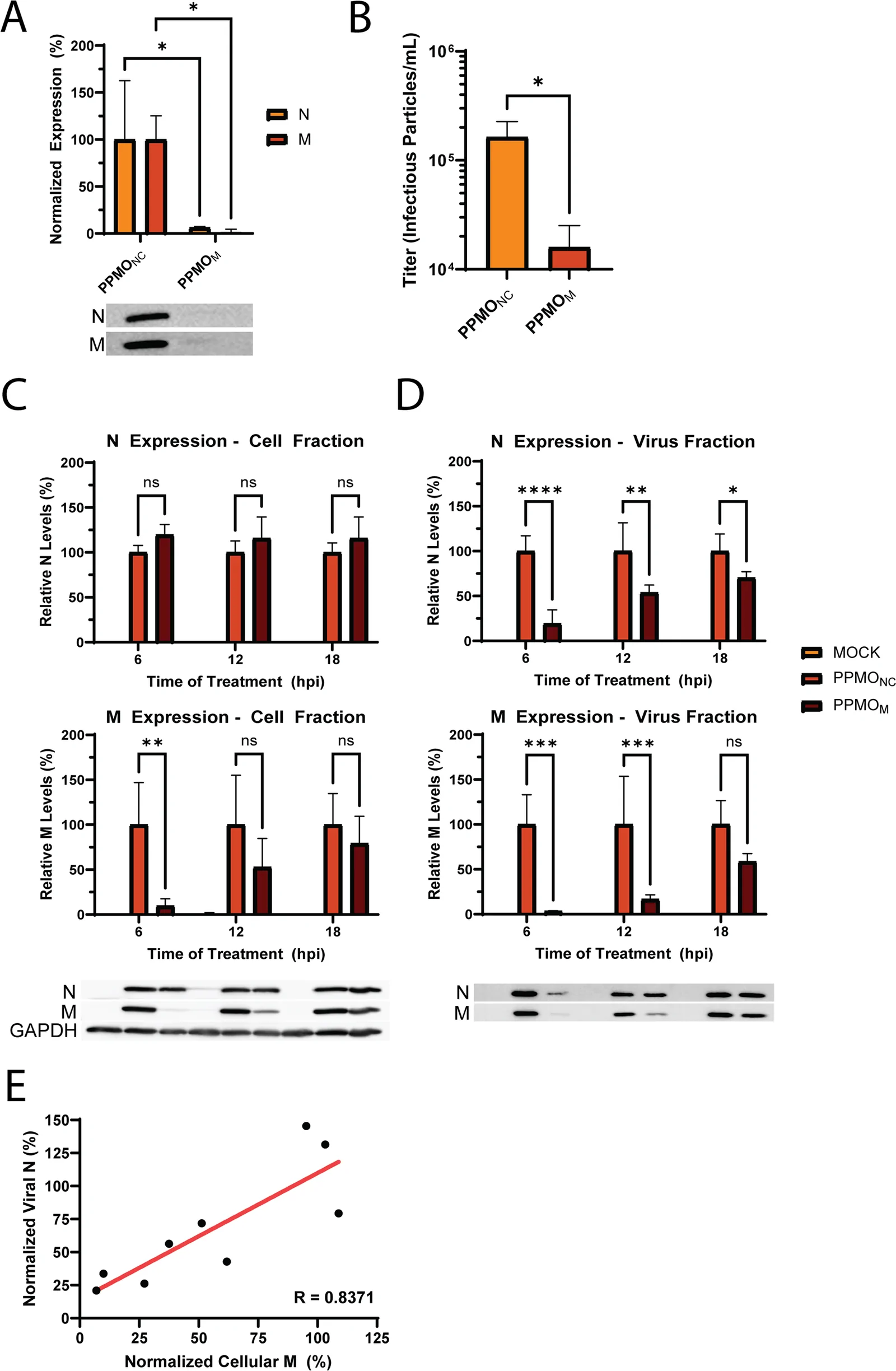
HMPV M protein levels directly correlate with viral particle production. A549 cells were MOCK-infected or infected with rgHMPV and treated with water, 10 µM PPMO_NC_, or 10 µM PPMO_M_ at 1 hpi. The infection was allowed to progress until 24 hpi, at which point the cultures were treated with 0.3 µg/mL TPCK-trypsin for 1 h. Purified virus was detected by immunoblotting with (**A**) anti-M and anti-HMPV N antibodies, or (**B**) particles were titered by immunofluorescence. Blots were quantified by densitometry and normalized to the PPMO_NC_-treated infection. (**C**) A549 cells were MOCK-infected or infected with WT HMPV and treated with 10 µM of PPMOs at 6, 12, or 18 hpi. After treatment, the infection was allowed to progress until 24 hpi, at which point cell lysates and (**D**) purified virus were immunoblotted with anti-HMPV N, anti-M, and anti-GAPDH antibodies. Blots were quantified by densitometry and were normalized to the PPMO_NC_-treated infection. (**E**) A549 cells were infected with WT HMPV and treated with 1, 5, or 10 µM of PPMOs at 1 hpi. At 24 hpi, free virus was purified from the supernatant, and the cells were lysed and used for immunoblotting with anti-HMPV N, anti-M, and anti-GAPDH antibodies. Blots were quantified by densitometry and were normalized to the PPMO_NC_-treated infection for each treatment concentration within each replicate and plotted (*n* = 3). ns, not significant, *P < 0.05; **P < 0.01; ***P < 0.001; ****P < 0.0001, by paired *t* test or two-way ANOVA.

A decrease in vRNP localization at the plasma membrane of PPMO_M_-treated cells suggests that fewer viral proteins are trafficked to assembly sites, potentially inhibiting viral budding. The M protein has been shown to be involved in virus assembly for multiple NNSVs, and HMPV M has been reported to be important for viral particle formation (76). To further detail the role for the HMPV M protein in viral particle formation, we quantified the amount of M and N released in the supernatant. Supernatants of infected and PPMO-treated cultures were collected, clarified, and purified on a 20% sucrose cushion. The purified virus was then lysed and used for immunoblotting. Treatment with 10 µM PPMO_M_ resulted in a 98.4% reduction in released M protein relative to PPMO_NC_ treatment (Fig. 7A), consistent with the suppression of M protein synthesis. Quantification of the HMPV N protein in the supernatant was used as a correlate for viral particle production, which showed a 93.8% reduction in N levels upon PPMO_M_ treatment when compared to PPMO_NC_ (Fig. 7A), confirming a critical function for the HMPV M protein in viral particle formation.

To assess how M protein expression affects viral titer, the recombinant GFP-expressing HMPV (rgHMPV)-infected culture was treated with TPCK-trypsin for 1 h, and the supernatants were then collected. A549 cells were then infected with serially diluted supernatant and incubated for 24 h, after which GFP-expressing cells were counted. The viral titers from cells treated with PPMO_M_ decreased by 10.3-fold relative to PPMO_NC_ treatment (Fig. 7B). Interestingly, there was still some viral particle production, suggesting that either low levels of the M protein are sufficient for small amounts of virus production or that other viral proteins are sufficient to drive virus production at low levels. Together, this confirms that M protein expression affects viral titers and that immunoblotting viral protein levels provides an effective method for approximating virus production.

An advantage of employing PPMOs is that they can be administered at specific times before or after infection, enabling investigations into time-dependent changes in protein levels during the course of an infection. Therefore, we administered PPMOs at early, middle, and late stages of infection. WT HMPV-infected cells were treated with 10 µM PPMOs at 6, 12, and 18 hpi to reduce M protein expression at different stages of infection. At 24 hpi, the M protein level was reduced to 9.6%, 52.8%, and 79.1% of PPMO_NC_ control levels for the 6, 12, and 18 hpi PPMO_M_ treatments, respectively (Fig. 7C). Infected cells treated with PPMO_M_ at these time points had slightly elevated N protein expression levels relative to PPMO_NC_ (119.6%, 115.7%, and 115.7% for 6, 12, and 18 hpi, respectively).

Viral particle formation was examined by measuring the amount of intracellular and extracellular N and M proteins present. Our data showed that the relative abundance of the M protein in cells correlated with the amount of the M and N proteins in the supernatant (Fig. 7C and D). The relative amount of N protein in viral particles was 19.3%, 53.7%, and 70.2% for 6, 12, and 18 hpi treatments, respectively (Fig. 7D). This suggests that M protein levels correlate directly with the degree of viral particle formation and that inhibiting M protein synthesis later in infection still has an impact on viral budding.

To assess if there was a direct correlation between the amount of the M protein present and the efficiency of viral particle release, infected A549 cells were treated with varying concentrations of PPMO at 1 hpi. The cells were then lysed, and the viral particles collected at 24 hpi were used for immunoblotting. Figure 7E shows a correlation plot of the cellular M and viral N protein levels. A strong correlation (R = 0.8371) between cellular M abundance and viral budding, as indicated by N protein levels, was observed. Together, these results showed that the M protein plays a significant role in viral budding and that M protein expression directly affects the number of viral particles formed.

### Decreased expression of HMPV M leads to changes in F distribution

**Fig 8:**
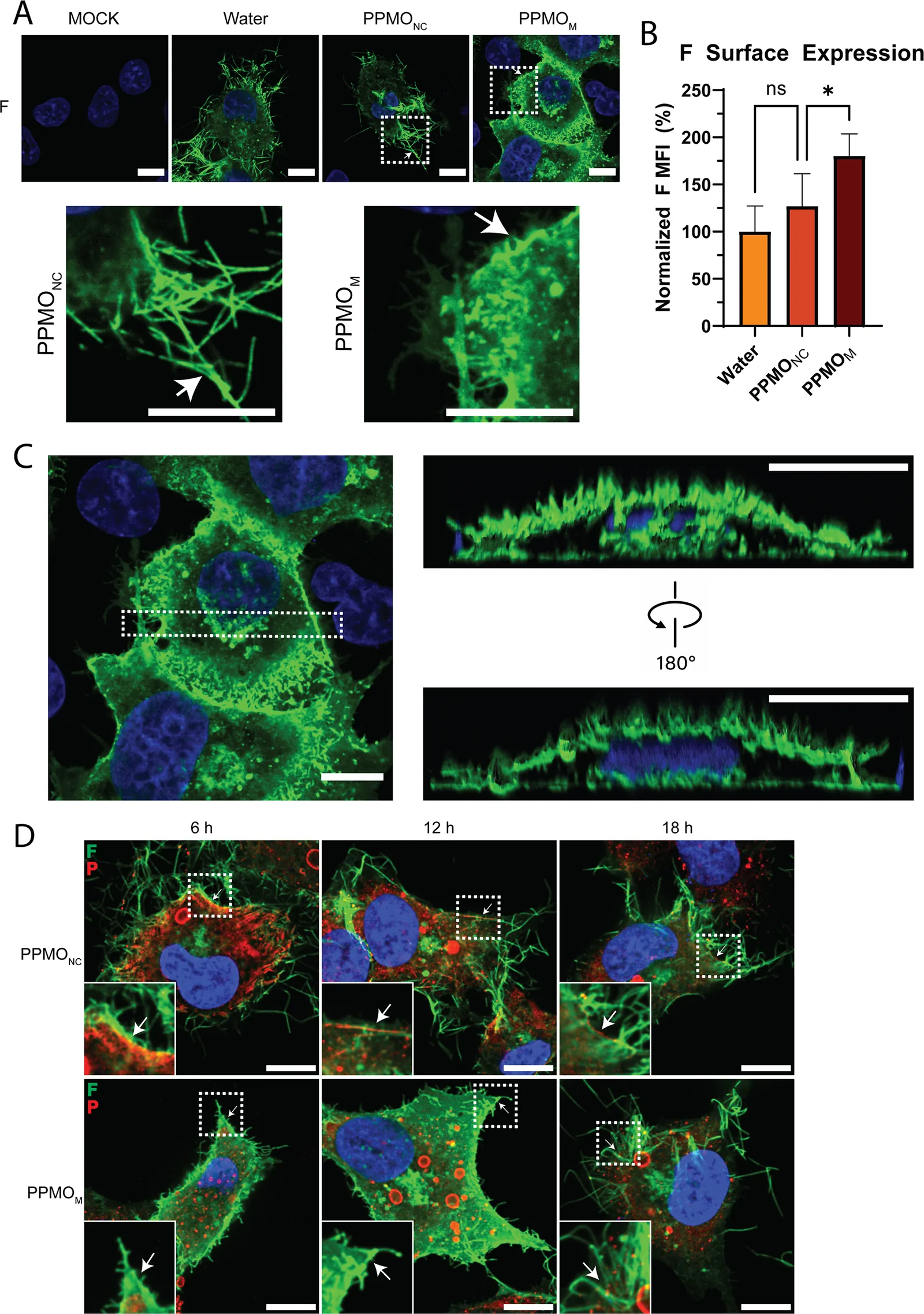
Decreased expression of HMPV M leads to changes in F distribution. A549 cells were infected with WT HMPV or MOCK-infected and then treated with water, 10 µM PPMO_NC_, or 10 µM PPMO_M_ at 1 hpi. At 24 hpi, (**A**) the cells were fixed and stained with anti-HMPV F (green) and DAPI (blue), followed by confocal microscopy. Magnified images are outlined by a white box. A white arrow points to mature and immature filaments. (**B**) Cells were stained for flow cytometry with an anti-HMPV F antibody and gated using F as a marker for infection. The mean fluorescence intensity (MFI) of F on the surface was measured by averaging the F signal intensity per infected cell and normalized to vehicle treatment. Data are shown as the mean ± SD (*n* = 4). (**C**) An image previously shown was cross-sectioned (shown by the dashed box) to show F protein (green) localization with respect to the nucleus stained with DAPI (blue). (**D**) Immunofluorescence imaging of A549 cells that were infected with WT HMPV and treated with 10 µM of PPMOs at 6, 12, or 18 hpi. The infected cells were fixed at 24 hpi and stained for cellular nuclei with DAPI (blue), anti-HMPV P (pseudo-colored red), and anti-HMPV F (pseudo-colored green) and imaged by confocal microscopy. The magnified regions are outlined in a white box, and arrows point to the edge of the cell. Data are from three independent experiments, and a single representative experiment is shown. Scale bars are 10 µm; ns, not significant, *P < 0.05 by one-way ANOVA.

Our experiments above showed that when M was reduced, a decrease in viral particle production (Fig. 7) and viral filament formation was observed (Fig. 3A). Therefore, we investigated the localization of the F protein upon M reduction. As observed previously (Fig. 3), viral filament formation was inhibited when the M protein was reduced (Fig. 8A). The filaments on the plasma membrane in the controls (shown by a white arrow) are consistent with virus budding from the plasma membrane and are called mature viral filaments. Under PPMO_M_ treatments, the filaments appeared smaller than those in the control infections (shown by a white arrow) (Fig. 8A). We termed these viral filaments immature because they are not fully developed into long viral filaments with increased F protein localization. This result demonstrated a key role for the M protein in viral filament maturation. Interestingly, F puncta were observed when M was reduced. Similar structures have been observed in RSV infection in the absence of the M protein (36). Together, these results suggest that the M protein is not required for the initiation of virus-induced filaments but that filament maturation is impeded when the M protein is reduced (36).

PPMO_M_-treated cells also showed F accumulation on the cell membrane of infected cells and localization in the perinuclear area. To confirm these results, we analyzed F protein surface expression using flow cytometry. PPMO_M_-treated cells had a 1.42-fold increase in F surface expression compared to PPMO_NC_-treated infections (Fig. 8B). Viral glycoproteins are synthesized in the endoplasmic reticulum (ER) and shuttled through the secretory pathway (76–78). To assess whether F indeed localizes on the cellular membrane and perinuclear region, confocal cross-sections were made. A representative cross-section shows clear localization of the F protein on the plasma membrane and in the perinuclear region (Fig. 8C), likely correlating with the ER and Golgi since F is a transmembrane protein (79) (Fig. 8C). Interestingly, there are variations in F intensity at the top of the cell, which may correlate with short viral filaments harboring increased F localization, similar to what was observed in RSV by Mitra et al. (36) (Fig. 8C).

To assess the effect of M protein reduction on viral protein localization at different time points during infection, PPMO_M_ was added at 6, 12, or 18 hpi, and the cells were fixed at 24 hpi. Blockage of new M protein synthesis starting at 6 hpi led to a near-complete loss of long viral filaments at 24 hpi, as shown by F staining (Fig. 8D). Treatments at 12 hpi also resulted in very few long external filaments, while infected cells treated at 18 hpi had a similar number of filaments to the PPMO_NC_ control (Fig. 8D). These results are consistent with the understanding that viral filament formation is initiated as early as 12 hpi and indicates that a sharp reduction in M protein synthesis at this time impedes further filament formation. Alternatively, efficient production of mature filaments could require more than ~50% of the WT M protein levels at 24 hpi because at the 18 hpi treatment, when total M protein levels were reduced by only ~21%, infected cells still possessed long viral filaments (Fig. 8D). This suggests that sufficient M protein levels have already been synthesized by 18 h for filament production and that the available pool of M was not depleted to a level where filament maturation ceases.

To investigate vRNP localization and further characterize any vRNP-associated changes observed with reductions in M protein expression, we used a nanobody targeting the HMPV P protein that was previously developed by our laboratory (47). Interestingly, P protein localization differed, depending on the time of PPMO_M_ treatment (Fig. 8D). When HMPV-infected cells were treated at 6 and 12 hpi, an increase in IB number was observed (Fig. 8D), consistent with our previous findings (Fig. 2B). However, the total number of IBs returned to control-like levels in infected cells treated at 18 hpi. Interestingly, PPMO_M_ treatment at 18 hpi yielded a heterogeneous infected-cell population. Some cells had P localized in filament-like structures on or near the plasma membrane, and some cells did not (shown by white arrows). These structures were readily observed with PPMO_NC_ treatment. This suggests that P protein localization may be more sensitive to changes in M protein levels at later time points during infection. Overall, this shows that inhibiting M protein expression at different stages of infection reveals different effects and elucidates a critical role for the M protein in driving viral filament maturation and viral protein localization.

## DISCUSSION

The majority of research on the M protein of viruses in the order *Mononegavirales* has focused on viral budding, with less understanding of other potential functions (29, 30, 32, 68, 80–82). Recombinant viruses or transfection-based systems have furthered our understanding of the M protein in these respective processes. However, these systems have a limited ability to investigate the M protein at different stages of infection. Therefore, we designed a PPMO to target HMPV M protein expression at various time points during an infection. We show that the HMPV M protein is the main driver of viral budding and that viral titers depend on M protein expression (Fig. 7). To our knowledge, this is the first report to systematically examine how varying reductions in M expression and the timing of M reduction can alter viral budding. We examined the M protein and budding in two ways with PPMOs: by initiating treatment at various time points post-infection, and by administering varying dose levels. By administering doses of PPMOs at specific times, we saw a change in total levels of M within cells, which correlated with the amount of the N protein found in viral particles (Fig. 7D). By administering various PPMO doses at 1 hpi, we observed a dose-dependent change in budding that correlated with M protein abundance in cells (Fig. 7E). Immunofluorescence imaging showed that PPMO treatments at 6 and 12 hpi reduced mature viral filaments at 24 hpi (Fig. 8D). The 12 hpi treatment condition reduced M expression by ~50% and a 5 µM PPMO_M_ treatment also showed less N localization in filaments (Fig. 2B and 7C). These results suggest that HMPV may need more than 50% of wild-type M levels to induce typical viral filament maturation. The literature shows that viral particles possess an M-lattice beneath the viral membrane (17, 83, 84). Therefore, it is possible that HMPV needs sufficient levels of the M protein to form the M-lattice and induce budding. We hypothesize that in instances where the M protein is significantly reduced, the M lattice may never fully form or may form at a considerably slower rate, leading to a reduction in viral particle formation that we observed. These results support that the M protein is critical for budding and that without it there is a substantial loss of viral particle production.

Our data showed that when the HMPV M protein was reduced by more than 95%, there was a significant loss of mature viral filaments (Fig. 8A). We also observed a reduction in P protein localization at the plasma membrane of infected cells when M was reduced. This showed that the M protein plays a role in P localization in infections (Fig. 5D and E). The literature has demonstrated that the HMPV P protein can induce membrane deformations (85) and also that RSV M and P proteins can interact with actin (86–88). Therefore, we hypothesize that during HMPV infection, the M protein recruits the P protein to assembly sites and alters actin architecture. Altogether, more research is needed to investigate how the interactions between M and P drive viral filament maturation.

In addition to changes in filament formation, the M protein likely plays a role in localizing the F protein. Our data showed that with PPMO_M_ treatment, the F protein was observed in immature filaments and puncta that are dispersed across the membrane of infected cells (Fig. 8A). In the vehicle or PPMO_NC_-treated infections, F concentrates in mature viral filaments and has little localization to other regions on the plasma membrane (Fig. 8A). This suggests that the M protein is directly or indirectly involved in coordinating F localization at the plasma membrane. This could be due to interactions with lipids or cellular/viral proteins that drive F localization into puncta. Previous studies have shown that the RSV M protein has more localization in IBs when the C-terminal tail of the RSV F protein is truncated (22, 89), suggesting the RSV M protein relies on F for proper localization. It is not known whether this is a direct or indirect interaction, but it does suggest that these two proteins coordinate the localization of each other through an interaction with the cytoplasmic tail of F. Therefore, further investigations are needed to elucidate the impact of M and F on assembly.

PPMO_M_ treatment to reduce M led to an increase in IB count, but a decrease in volume (Fig. 3B), which differs from a study in which parainfluenza virus type-3 (PIV3) proteins were transiently expressed in cells (68). In that study, which used a transfection-based expression system to measure changes in IB size, more M led to smaller IBs. To assess whether the differences were due to infection versus transfection expression, we conducted a transfection-based study with HMPV proteins. Interestingly, transient transfection-based expression with variations in HMPV M expression showed no change in IB size (Fig. 4D). Our transfection-system results also differed from those observed during HMPV infection with M expression knockdown, where changes in IB size were observed (Fig. 4D). This suggests that the presence of other viral proteins, such as F or L, may be important for the observed effect of M changes on IBs. Prior investigations with M-null RSV showed similar numbers of IBs when compared to a control infection, but the average area of IBs had doubled (36). There may be differences in the M protein between *pneumoviruses* and *paramyxoviruses*, and the very small amount of M remaining in our system may lead to the different observations between RSV and HMPV. However, for HMPV, we showed that when the M protein is depleted, vRNA localization is impacted.

Previous work studying PIV3 suggested that the M protein reduces vRNA synthesis through an N-M interaction (68). Additional work with RSV provided evidence of an interaction between the M2-1 and M proteins (35), although this finding was not observed by others (23). Nevertheless, these previous investigations demonstrate that the M protein may play a role in changing the polymerase function by sequestering viral proteins involved in viral replication. Our results showed no change in total vRNA levels, transcripts, or replication products when the M protein was reduced by 95% (Fig. 4). This suggests that the HMPV M protein does not directly affect viral genome replication or transcription during infection, possibly due to differences in the proteins M interacts with between viruses.

**Fig 9:**
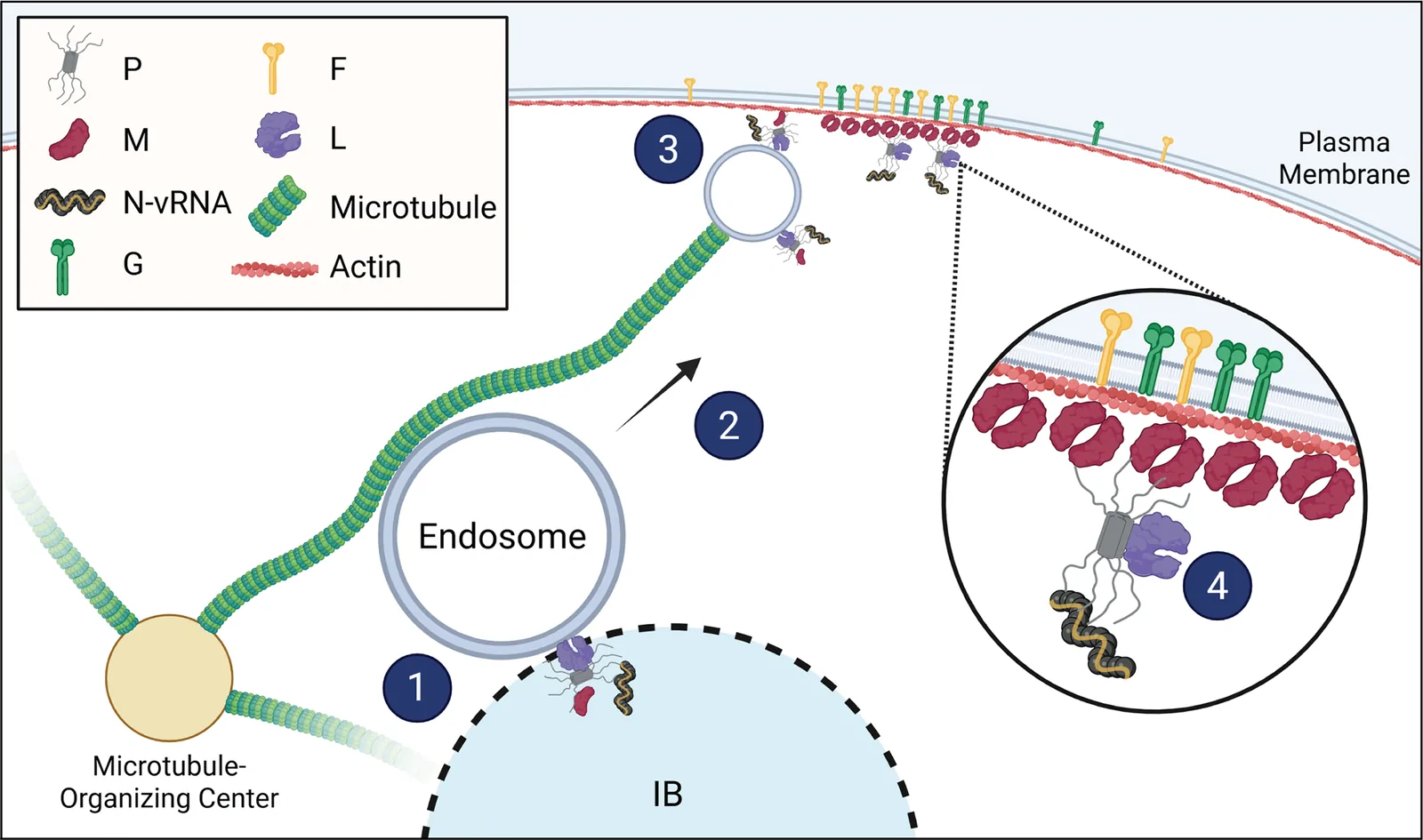
Proposed mechanism for the M protein in viral ribonucleoprotein localization. Model for the HMPV M protein in vRNP localization. (1) The viral L (purple), N-RNA (black/yellow), P (gray), and M proteins (red) are recruited onto endosomes at the IB periphery. (2) The vRNPs are then trafficked with the movements of endosomes on microtubules (green) until they reach the plasma membrane. (3) Viral RNPs become associated with the plasma membrane through an M interaction with either cortical actin (red), the plasma membrane, or the cytoplasmic tails of the F (yellow) or G (green) proteins. (4) The M-vRNP complex remains associated with the plasma membrane in the absence of endosomes.

We observed an increase in IB count and a decrease in size when the M protein is reduced by approximately 95% (Fig. 3B). These changes were observed at both 12 and 24 hpi, demonstrating that this phenotype is not due to the accumulation of viral proteins when budding is reduced by PPMO_M_ treatment. Our data also showed that vRNPs have decreased colocalization with the plasma membrane, but that they co-traffic with M (Fig. 5). These results support the model where M and vRNPs traffic on endosomes until they reach the cell periphery, where the key role of the M protein in vRNP trafficking is to stably transfer vRNPs to the plasma membrane region (Fig. 9). Our work taken together with previously published studies suggests a complex model for transport of components to the assembly site, with HMPV M playing a critical role at the end of the process (Fig. 9). We hypothesize that the HMPV P protein scaffolds M, N-vRNA, and L to form a transport complex. Previous research has shown that the RSV M protein interacts with the RSV P protein (23) and that the C-terminal tail of the HMPV P protein interacts with the HMPV N-RNA complex (90). The L protein has also been shown to interact with P for a few negative-stranded RNA viruses (91–93). Therefore, the P protein may interact with M, N-RNA, and L simultaneously to form a transport complex. The RSV L protein has been shown to interact with Rab11a (74); hence, L can recruit the entire M-vRNP complex onto Rab11^+^ endosomes. Our data showed that M protein reductions do not impact intracellular vRNP transport (Fig. 6), consistent with the recent report that the RSV L-Rab11a interaction is critical for recruitment of vRNPs onto endosomes (74). Together, this suggests that the M protein is not required to recruit vRNPs onto endosomes or for their movement within cells. However, our data suggest that M is necessary for stable transfer of vRNPs to the plasma membrane (Fig. 5E). If M is reduced, vRNPs may traffic back into the cytoplasm, dissociate from the endosome, and form a new IB, thus aligning with our observations of increased IB numbers (Fig. 3). Therefore, this data highlights a critical role for the M protein in stably transferring the vRNPs to the plasma membrane (Fig. 9). This work adds important new information to understand the function of the M protein in HMPV infection.

## Data Availability

All data supporting the findings of this study are available upon reasonable request from the corresponding author.

## SUPPLEMENTAL MATERIAL

Supplemental material jvi.01066-26-s0001.docx Fig. S1 to S5.

ASM does not own the copyrights to Supplemental Material that may be linked to, or accessed through, an article. The authors have granted ASM a non-exclusive, world-wide license to publish the Supplemental Material files. Please contact the corresponding author directly for reuse.
