# Supplementary material for: Reduced expression of human metapneumovirus matrix protein impacts virus assembly and ribonucleoprotein localization
Source: J Virol. 2026 Aug 31;100(9):e01066-26. doi: 10.1128/jvi.01066-26 (PMC13595917; doi:10.1128/jvi.01066-26)
Supplement: Supplementary file 1 [file jvi.01066-26-s0001.docx]

*Supplementary Figures*

Supplementary Figure 1

*HMPV genes have a high degree of sequence conservation in the 5’ UTRs of mRNAs.* The HMPV CAN 97-83 genome was analyzed for sequence conservation around the initiator-AUG codon (shown in red) of eight genes. 25 and 22 nucleotides were analyzed before and after the AUG codon, respectively. Nucleotides that are at least 50% conserved are shaded in shades of violet, with the darker shades being more conserved. White areas show nucleotides that are not well-conserved across the eight genes.

Supplementary Figure 2

*PPMOs do not impact cell viability at a 10 µM concentration.* A549 cells were treated with 10 µM of PPMOs. At 24 hours post-treatment, the cells were stained with trypan blue to count the dead and living cells with a hemocytometer. The % viability was calculated by dividing the number of living cells by the total number of cells counted and then expressed as a percent. The data are from three independent experiments and are shown as the mean ± SD (n=3).

Supplementary Figure 3

*PPMOs modestly reduce infection rate.* A549 cells were infected with WT HMPV and treated with 10 µM of PPMOs at 1 hpi. At 24 hpi, the cells were washed and stained with an anti-HMPV F antibody and a secondary antibody conjugated with Alexa Fluor 488. The infection rate was quantified by measuring the number of infected cells by flow cytometry and dividing by the total number of cells counted, and expressed as a percentage. The data are from three independent experiments and are shown as the mean ± SD (n=3). ** p < 0.01, *** p < 0.001 by one-way ANOVA.

Supplementary Figure 4

*Treating with PPMOs at a later time restores infection rate.* A549 cells were infected with WT HMPV and treated with a non-specific PPMO_COVID-AUG_ at a 10 µM concentration at 1 or 6 hpi. At 24 hpi, the cells were washed and stained with an anti-HMPV F antibody and a secondary antibody conjugated with Alexa Fluor 488. The infection rate was quantified by measuring the number of infected cells by flow cytometry and dividing by the total number of cells counted, and expressed as a percentage. The data are from three independent experiments and are shown as the mean ± SD (n=3). ns, not significant, * p < 0.05, by one-way ANOVA.

Supplementary Figure 5

*mCherryP traffics with Rab11a^+^ recycling endosomes*. A549 cells were infected with WT HMPV and subsequently transfected with 1 µg GFP-Rab11a (green) and 1 µg mCherryP (red) at 3 hpi. At 24 hpi, live cells were imaged. Shown is a representative single z-slice time lapse (n=3). The white arrows show an RNP complex moving throughout the cell with GFP-Rab11a. Scale bar is 1 µm.
